# A Comparative Study of Doppler‐Guided Acute Bone Shortening‐Lengthening Versus Bone Transport for Infected Tibial Defects Combined With Soft Tissue Defects

**DOI:** 10.1111/os.70409

**Published:** 2026-08-26

**Authors:** Zhiming Zhao, Guoqi Ji, Chengkuo Cai, Weiguo Xu

**Affiliations:** ^1^ Department of Traumatic Orthopaedics, Tianjin Hospital Tianjin University Tianjin China

**Keywords:** bone lengthening, bone transport, external fixators, soft tissue defect, tibial defect

## Abstract

**Objective:**

The management of infected tibial bone defects with soft tissue defects (ITBD‐STD) remains challenging. This study aims to establish a Doppler‐guided safety threshold for acute shortening (≤ 3 cm), compare the clinical efficacy and safety of shortening‐lengthening (S‐L) versus bone transport (BT), quantify the complication burden, and clarify the calculation indices.

**Methods:**

A retrospective analysis was conducted on the clinical data of 43 patients with ITBD‐STD admitted between January 2021 and January 2024. All patients were treated using the Ilizarov circular external fixation technique and were divided into a bone S‐L group and a BT group based on the surgical procedure. In the S‐L group, acute shortening was performed under real‐time Doppler guidance with a predefined safety cap of 3 cm. The wound healing time (WHT), bone healing index (BHI), external fixation index (EFI), and complication rate were compared and analyzed between the two groups. At a mean follow‐up of 19.9 months, bone union and lower limb functional recovery were evaluated using the criteria of the Association for the Study and Application of the Ilizarov Method (ASAMI). Statistical analysis was performed using the independent sample *t*‐test or the *χ* ^2^ test.

**Results:**

The WHT in the S‐L group was significantly shorter than that in the BT group (68.5 ± 25.2 vs. 85.2 ± 21.5 days, *p* < 0.05). In the S‐L group, acute shortening was successfully performed in all 22 patients under real‐time Doppler guidance, with a mean acute shortening distance of 2.8 ± 0.4 cm (capped at 3 cm). No patient experienced Doppler signal loss or required intraoperative termination of shortening due to vascular compromise. The BHI (37.36 ± 10.73 vs. 44.14 ± 11.12 days/cm) and EFI (45.36 ± 11.23 vs. 53.52 ± 12.55 days/cm) in the S‐L group were significantly lower than those in the BT group (all *p* < 0.05). However, there was no statistically significant difference in the overall complication rate between the two groups (45.5% vs. 42.9%, *p* > 0.05). At the final follow‐up, there was also no statistically significant difference in ASAMI scores between the two groups (*p* > 0.05).

**Conclusion:**

Both S‐L and BT are effective for ITBD‐STD with comparable safety. Under the strict Doppler‐guided protocol (≤ 3 cm), S‐L significantly shortens WHT without increasing complications, yet this benefit is design‐inherent rather than a biological advantage. Given comparable ASAMI functional outcomes, S‐L offers no functional superiority over BT. Nonetheless, the Doppler criteria and selection strategy established herein provide a safe, reproducible framework for technique selection, validating the clinical feasibility of acute shortening without compromising final function or safety.

## Graphical abstract

Under a validated Doppler‐guided safety protocol (≤ 3 cm), acute shortening‐lengthening and bone transport show comparable safety and functional outcomes for infected tibial defects, but shortening‐lengthening significantly reduces wound healing time—a design‐inherent benefit without functional superiority.

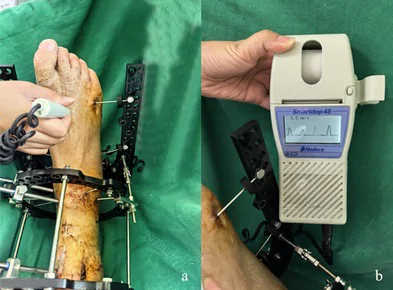

## 1 Introduction

Infected tibial bone defects with soft tissue defects (ITBD‐STD) remain a highly challenging problem in the field of orthopedic reconstruction [1]. This condition is usually caused by high‐energy trauma, postoperative deep infection, or debridement for chronic osteomyelitis, resulting in significant bone loss, impaired soft tissue coverage, and persistent infection. Effective management requires multidisciplinary collaboration to simultaneously address infection eradication, bone reconstruction, and soft tissue coverage to restore limb function and avoid amputation [2]. External fixation systems, especially circular fixators utilizing the principle of distraction osteogenesis, have become the primary means of treating such diseases because they provide stable fixation while promoting bone lengthening or transport [3, 4].

The Ilizarov bone transport (BT) technique is widely recognized as the “gold standard” for treating segmental bone defects. This method allows for the gradual regeneration of new bone at the osteotomy site while transporting the bone segment to the docking site. However, this method is often accompanied by a long treatment cycle, a high incidence of nonunion at the docking site, and a heavy burden on patients during the prolonged treatment period [5]. On the other hand, the shortening‐lengthening (S‐L) technique—involving acute shortening followed by gradual lengthening—has gained attention as a potential alternative, particularly for infected tibial defects. This method aims to create conditions for direct soft tissue closure or simplified coverage surgery by acutely shortening the defect, thereby potentially reducing the EFI and the risk of pin tract infection [6, 7].

A major constraint of the S‐L technique is the safety limit of acute shortening, primarily dictated by the risk of neurovascular compromise. Traditionally, intraoperative assessment of limb perfusion during acute shortening relies on subjective clinical indicators such as capillary refill, skin color, and palpable pulses. These methods are often unreliable, especially in the setting of ITBD‐STD where chronic infection and prior surgeries have already compromised local soft tissue and microcirculation. This unreliability forces surgeons to be overly conservative, limiting the degree of shortening, or poses a catastrophic risk of limb ischemia if shortening exceeds vascular tolerance. While intraoperative Doppler ultrasound has been utilized to monitor vascular flow in other orthopedic reconstructive procedures, such as limb lengthening for congenital or post‐traumatic deformities [8], its application as a real‐time guidance tool specifically during acute shortening for infected tibial defects remains rarely reported. Previous comparative studies have only vaguely mentioned “observing circulation” or “without neurovascular compromise” without providing explicit, objective monitoring protocols or defined safety thresholds [7]. Establishing an objective, real‐time Doppler‐guided safety threshold is thus critical to maximize the benefits of acute shortening while eliminating the risk of vascular catastrophe. However, no prior study has provided such an explicit Doppler‐guided protocol specifically for infected tibial defects, nor a transparent strategy to guide technique selection. This knowledge gap forms the primary impetus for the present investigation.

Beyond the Doppler‐specific gap identified above, the optimal strategy for ITBD‐STD remains controversial. BT is associated with a prolonged treatment course and considerable complications [9, 10], whereas acute shortening raises unresolved concerns about vascular safety and the degree of shortening [11]. These concerns stem largely from the lack of an objective, safe protocol, as traditional intraoperative assessments—capillary refill and pulse palpation—are inherently subjective. Such methods are particularly unreliable in infected tibial defects, where soft tissue edema, prior surgical scarring, and microvascular damage frequently render pedal pulses nonpalpable. Several comparative studies have reported that S‐L shortens treatment duration compared with BT [7, 12, 13]. However, these studies primarily relied on ratio indices (BHI, EFI) without providing explicit criteria for technique selection or quantifying the clinical burden of complications beyond categorical reporting. To address these gaps, this retrospective study of 43 patients with ITBD‐STD aims to: (i) establish explicit, Doppler‐guided criteria for acute shortening (capped at 3 cm) and clarify the clinical rationale driving technique selection between S‐L and BT; (ii) compare the two techniques with respect to wound healing time (WHT), bone healing index (BHI), external fixation index (EFI), complication rates, and ASAMI functional scores; and (iii) quantify clinical burden using reoperation rates and additional hospital days.

## 2 Materials and Methods

### 2.1 General Information

This study retrospectively analyzed the clinical data of 43 patients with ITBD‐STD admitted to the Department of Orthopedic Traumatology at Tianjin Hospital, Tianjin University from January 2021 to January 2024. All patients were treated using the Ilizarov circular external fixation technique and were divided into the S‐L group (22 cases) and the BT group (21 cases) based on the surgical procedure. All surgeries were performed by the same team of orthopedic trauma surgeons. This study was approved by the Ethics Committee of Tianjin Hospital, Tianjin University (approval no. 2026‐002) and was conducted in accordance with the principles of the Declaration of Helsinki. Written informed consent was obtained from all patients.

Patients were assigned to either the S‐L group or the BT group based on the surgical procedure actually performed. The decision was made intraoperatively by the attending surgeon according to the following principles: S‐L was chosen when acute shortening ≤ 3 cm could achieve direct or near‐direct soft tissue closure without compromising distal perfusion (confirmed by Doppler); BT was chosen when acute shortening was deemed unsafe (e.g., estimated residual perfusion compromise, excessive nerve tension, or anticipated major limb length discrepancy [LLD]), or when the patient declined the expected shortening. No formal randomization was applied.

Inclusion criteria: (i) age range of 19–65 years; (ii) patients with traumatic ITBD‐STD; (iii) those who underwent S‐L or BT using the Ilizarov technique during treatment; (iv) bone defect length between 3 and 10 cm; (v) postoperative follow‐up time ≥ 18 months. Exclusion criteria: (i) fracture line involving the tibial articular surface and lacking a tibial segment available for bone transport; (ii) combined with severe dysfunction of vital organs such as the heart, lung, liver, or kidney, making the patient unable to tolerate anesthesia or surgery; (iii) poor compliance, unable to tolerate long‐term external fixation treatment; (iv) incomplete follow‐up data.

### 2.2 Preoperative Preparation

Preoperative optimization strategies are crucial for ensuring surgical safety and efficacy. Medical risk stratification requires multidisciplinary collaboration to systematically assess the patient's general condition and identify absolute surgical contraindications. Meanwhile, wound secretions should be collected for bacterial culture and drug susceptibility testing to guide targeted antibiotic use. Additionally, single‐photon emission computed tomography (SPECT/CT) is recommended for the precise assessment of the extent and severity of bone and soft tissue infections, providing an objective basis for surgical planning. These comprehensive measures contribute to comprehensive preoperative risk management and accurate assessment of infection status.

### 2.3 Surgical Technique

The selection of S‐L versus BT was determined after completion of debridement and measurement of the bone and soft tissue defects, following the criteria described in Section 2.1.

#### 2.3.1 Debridement

Under general anesthesia or epidural block anesthesia, the patient was placed in the supine position, and a tourniquet was routinely applied to the affected limb. For patients with a history of surgery or accompanying soft tissue defects, debridement was performed via the original approach or the soft tissue defect site, with layer‐by‐layer dissection to the lesion site, minimizing damage to the local blood supply. If internal implants were present, they were carefully removed. Subsequently, infected and necrotic bone and soft tissues at the stumps were thoroughly debrided, sinus tracts were excised, and the medullary cavity was opened. Three deep soft tissue specimens from different parts of the wound cavity were collected for bacterial culture and drug susceptibility testing. The wound was repeatedly soaked with hydrogen peroxide and iodophor, and the wound cavity was repeatedly irrigated using a pulse device on alternating high and low settings. An oscillating saw was used to shorten and trim both ends of the tibial defect; the extent of shortening was primarily determined by the achievement of satisfactory soft tissue coverage at the bone defect site.

#### 2.3.2 BT Group

The circular external fixator was pre‐assembled (CareFix Frame software, Version 2.1, Shanghai CareFix Pharmaceutical Technology Co. Ltd., China). Three fixation units were generally selected for the external fixator, installed at the proximal and distal ends of the osteotomy plane and on the transport bone segment. Each fixation unit typically contained 1–2 full rings, fixed with 2.0 mm olive wires (2 wires) and 1–2 6.0 mm hydroxyapatite half‐pins. When the lesion was close to the knee joint, a 2/3 ring was used; if accompanied by equinovarus deformity or to prevent equinovarus deformity, a U‐shaped ring was installed on the foot.

#### 2.3.3 S‐L Group

The external fixator was installed in the same manner as in the BT group. Fibular osteotomy was performed at the same level as the tibial bone defect; the osteotomy length was slightly longer than the tibial defect length, and the residual fibular segment was at least 6 cm above the ankle joint plane to ensure ankle stability. The initial intraoperative bone shortening generally did not exceed 3 cm; during the shortening process, a portable Doppler ultrasound device (Smartdop 45, Hadeco Inc., Japan) equipped with an 8‐MHz continuous‐wave probe was used in real‐time to monitor the flow signals of the dorsalis pedis artery and/or posterior tibial artery at the ipsilateral foot. The probe was placed at the standard anatomical landmarks, and the audible pulsatile signal was continuously monitored. Shortening was performed incrementally; if the Doppler signal became significantly weakened or disappeared, the maneuver was immediately reversed by 0.5 cm and held for 5 min for reassessment. The endpoint of safe shortening was defined as the maximum compression achievable while maintaining a clear, audible Doppler signal. All Doppler assessments were performed by the same two senior surgeons who had undergone standardized training on this protocol. Throughout the procedure, foot sensation and motor function were also closely monitored; any abnormality prompted immediate cessation of shortening and neurological reassessment. Bone lengthening was stopped when fluoroscopy showed that the affected limb was equal in length to the healthy side. The standardized Doppler monitoring protocol is illustrated in Figure 1.

**FIGURE 1:**
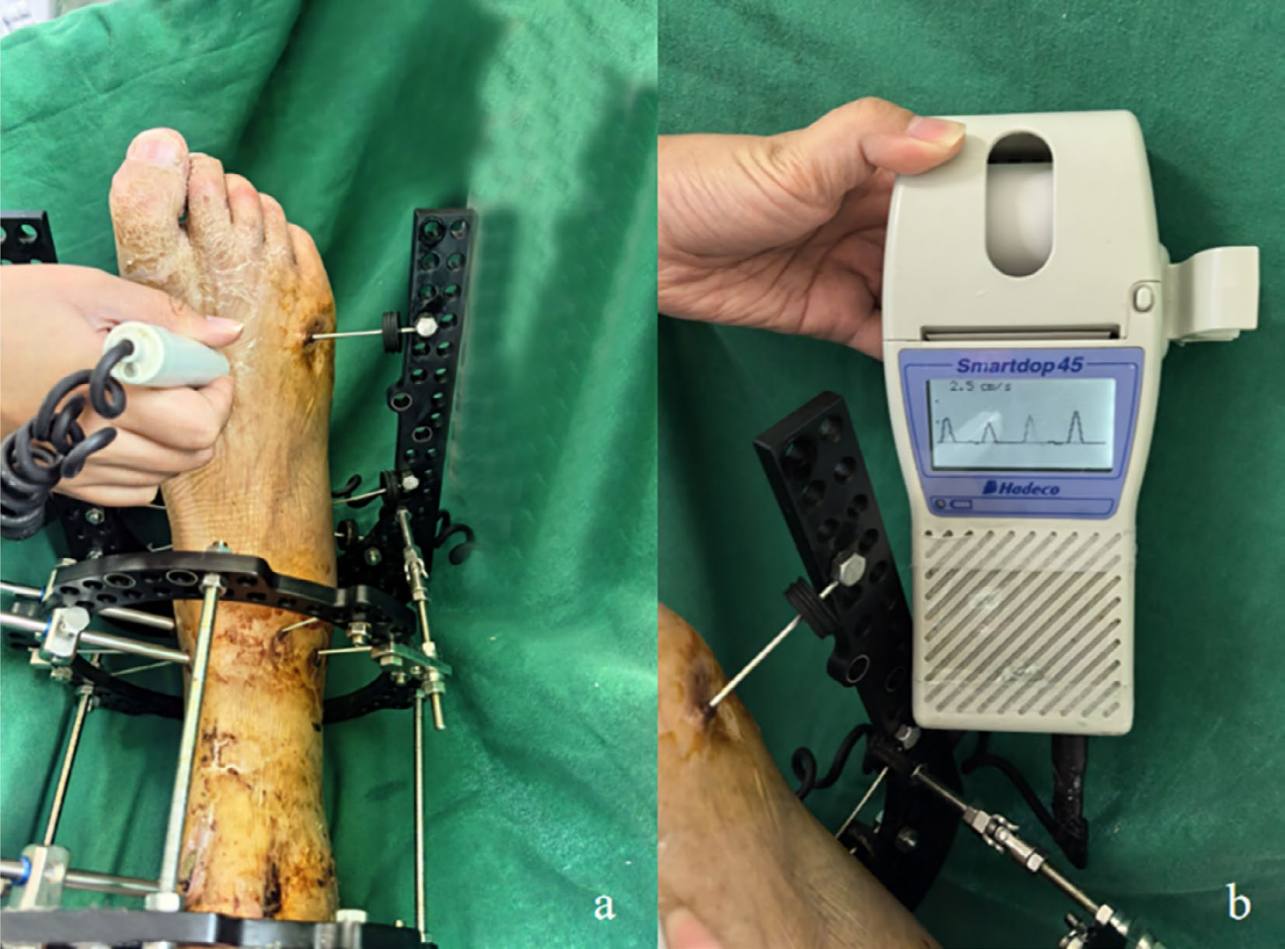
Intraoperative real‐time Doppler monitoring during acute shortening. (a) The portable Doppler probe is placed over the standard anatomical landmark of the dorsalis pedis artery on the ipsilateral foot. (b) The corresponding Doppler waveform shows a clear, uninterrupted arterial pulsatile signal at the maximal acute shortening point (up to 3 cm), confirming persistent distal perfusion.

#### 2.3.4 Osteotomy

The surgical personnel scrubbed again, disinfected the surgical area and the external fixator, draped sterile towels, and replaced surgical instruments. A longitudinal incision approximately 1 cm long was made anterior to the tibia centered on the osteotomy site. Layer‐by‐layer dissection was performed, and the periosteum was incised longitudinally; care was taken to protect the integrity of the periosteum and to retract it appropriately to both sides to expose the anterior tibial cortex. A 4.5 mm drill bit was used to drill multiple holes at the planned osteotomy plane. Subsequently, a 5 mm bone chisel was used to chisel through the medial and lateral tibial cortices; when the chisel reached the posterior cortex, a 14 mm hex wrench was used to assist in twisting until the cortex was completely fractured (or a fracture sound was heard). C‐arm fluoroscopy confirmed complete tibial transection. Finally, the bone ends were reduced to the pre‐osteotomy position and appropriately compressed using threaded rods.

#### 2.3.5 Postoperative Management

Antibiotic treatment was initiated based on preoperative bacterial culture results and switched to sensitive antibiotics after intraoperative bacterial culture and drug susceptibility results were returned. The pin tract was disinfected daily with 75% ethanol, and the wound cavity of the bone and soft tissue defect was disinfected with iodophor and packed with gauze. Functional exercises for the knee, ankle, and quadriceps femoris began on the 2nd day after surgery, and partial weight‐bearing of the affected limb under the protection of double crutches was performed on the 3rd to 4th day after surgery. BT was conducted at a rate of 0.25 mm per session, four times daily, on the 7th–10th day after surgery. If the external fixator did not span the ankle joint, a strap should be used to suspend and fix the ankle joint in the neutral position to prevent contracture.

For the S‐L group, if the bone defect site remained continuously exposed postoperatively, tibial shortening was continued at a rate of 1 mm per session, hree times daily, starting from the 3rd day after surgery. During treatment, Doppler ultrasound was used to strictly monitor blood flow in the dorsalis pedis and posterior tibial arteries until the defect site was completely covered by soft tissue, achieving primary wound healing or coverage via local flap transfer or free skin grafting over granulation tissue. Simultaneously, the nerve responses of the limb were closely monitored. Once sensory abnormalities or circulatory disturbances occurred, lengthening or shortening was immediately stopped, and the bone segment was retracted to the position of the previous day; shortening or lengthening was resumed the next day after sensation and blood circulation returned to normal.

In essence, a unified standardized protocol was applied across both groups: progressive shortening in the S‐L group was strictly capped by real‐time Doppler monitoring, while the BT speed in the BT group was individually titrated during monthly follow‐ups based strictly on radiological evidence of regenerate mineralization and mechanical axis alignment.

After discharge, all patients received monthly follow‐up. The mineralization of the new bone and the alignment of the fracture were assessed by periodically taking x‐rays, which guided the adjustment of the BT speed and the correction of the mechanical axis until the external fixator was removed. The removal of the external fixator required meeting strict radiological and clinical criteria simultaneously: in terms of radiology, anteroposterior and lateral x‐rays showed bridging callus in at least three cortices at the fracture site, indicating solid fracture healing; in clinical terms, the affected limb could fully bear weight, and the fracture site was painless without tenderness. The final removal decision was jointly confirmed by at least two senior surgeons based on recent imaging and clinical examination results.

#### 2.3.6 Evaluation

At the final follow‐up, bone healing and functional recovery were evaluated using the ASAMI scoring criteria [7]. Bone healing evaluation was based on four indicators: degree of bone healing, infection status, correction of angular deformity, and degree of lower LLD, with outcomes classified as excellent, good, fair, or poor. Functional recovery evaluation covered six indicators: knee and ankle range of joint motion, pain severity, limp status, local tissue nutritional status, and the degree of contracture and reduced mobility of the knee and ankle joints; outcomes were similarly classified as excellent, good, fair, or poor. Surgery‐related complications were tallied according to the Paley classification criteria [14], falling into three major categories: problems, obstacles, and sequelae. Problems refer to complications that do not require surgical intervention; obstacles refer to complications requiring surgical intervention; and sequelae refer to intraoperative injuries and unresolved complications remaining after the conclusion of treatment.

WHT was defined as the number of days from the index surgery (debridement and external fixator application) to the date when the soft tissue defect achieved complete epithelialization without any discharge, requiring no further dressing changes, regardless of whether healing occurred by primary closure, secondary intention, skin grafting, or local flap transfer. The same definition was applied uniformly to all patients in both groups. The denominator for BHI and EFI was defined as the total postoperative distraction distance (i.e., the original bone defect length), excluding any intraoperative acute shortening. This same approach was used for both groups. This denominator definition was adopted because previous studies have inconsistently specified whether acute shortening was included or excluded [7, 12, 13]. In the S‐L group, using the net postoperative distraction distance as the denominator would mathematically inflate the EFI and BHI, thereby underestimating the true efficiency of this technique. To avoid this bias and ensure a fair comparison under the same clinical endpoint (restoration of equal limb length), we uniformly defined the denominator as the original bone defect length for both groups. By treating the acute shortening distance as part of the total length deficit to be restored, this approach ensures that the EFI and BHI accurately reflect the overall treatment burden rather than merely the postoperative lengthening phase.

#### 2.3.7 Statistical Analysis

Statistical analysis was performed using SPSS version 22.0 (IBM Corp., USA). Normality of continuous data was confirmed using the Shapiro–Wilk test. All continuous variables (such as age, number of surgeries, bone defect length, soft tissue defect area, time from injury to final surgery, WHT, BHI, EFI, and follow‐up time) satisfied the assumption of normality; therefore, they were expressed as mean ± standard deviation (mean ± SD), and comparisons between groups were made using the independent samples *t*‐test. Nonnormally distributed variables would have been analyzed using the Mann–Whitney U test, but none were identified in this dataset. Categorical variables (such as gender, injury mechanism, location of bone defect, type of complications, and ASAMI bone and functional scores) were expressed as counts (percentages). Comparisons between groups were made using the *χ* ^2^ test, or Fisher's exact test when the expected frequency in any cell was less than 5. A *p* value < 0.05 was considered statistically significant.

Given the limited sample size, the study had insufficient statistical power to detect small‐to‐moderate differences in complication rates or to reliably confirm the equivalence of baseline characteristics between groups. Thus, the nonsignificant *p*‐values for baseline comparisons do not guarantee group comparability, and the complication‐related results should be viewed as exploratory.

## 3 Results

### 3.1 General Data

A total of 43 patients with ITBD‐STD were included in this study, including 22 cases in the S‐L group and 21 cases in the BT group. The follow‐up time of the two groups (S‐L group: 19.6 ± 5.5 months; BT group: 20.2 ± 6.8 months) showed no statistically significant difference *(p* > 0.05). The overall mean follow‐up time was 19.9 months (range: 18–24 months). The study cohort included 28 males and 15 females, with a mean age of 44 years (range: 19–65 years). There were no statistically significant differences in baseline characteristics between the two groups, including gender, age, injury mechanism, affected limb side, number of previous surgeries, location of bone defect, length of bone defect, area of soft tissue defect, and the time interval from injury to definitive treatment (all *p* > 0.05), indicating that no statistically significant differences in baseline characteristics were detected between the two groups. However, given the limited sample size, these non‐significant *p*‐values should not be interpreted as proof of group equivalence. The two groups were considered clinically similar with respect to the measured variables, but residual confounding cannot be excluded (Table 1).

**TABLE 1:** Baseline characteristics in the S‐L group and BT group.

Variable	S‐L group (*n* = 22)	BT group (*n* = 21)	*p*
Gender			0.747
Male	15	13	
Female	7	8	
Age (year)	43.4 ± 8.4	45.1 ± 8.5	0.515
Injury mechanism			0.879
Traffic accident	15	14	
Crush injury	4	5	
Fall from height	3	2	
Injured bone			0.768
Left tibia	12	13	
Right tibia	10	8	
Number of operations	2.1 ± 1.2	2.0 ± 1.5	0.815
Location of bone defect			0.914
Proximal tibia	1	1	
Tibial shaft	7	5	
Distal tibia	14	15	
Length of bone defect (cm)	6.2 ± 2.1	5.4 ± 2.4	0.262
Area of soft tissue defect (cm^2^)	36.1 ± 12.3	30.2 ± 11.2	0.104
Time elapsed since the injury To definitive treatment (month)	13.2 ± 4.3	12.8 ± 4.1	0.757
Follow‐up time (months)	19.6 ± 5.5	20.2 ± 6.8	0.752

Abbreviations: BT, bone transport; S‐L, shortening‐lengthening.

### 3.2 Peri‐Operative Conditions

Patients in both groups eventually achieved clinical bone healing at the distraction osteogenesis site and the docking site. After removal of the external fixator, all patients were able to achieve weight‐bearing walking. In the S‐L group, real‐time Doppler monitoring confirmed uninterrupted perfusion of both the dorsalis pedis and posterior tibial arteries in all 22 cases throughout the acute shortening procedure. The mean acute shortening distance was 2.8 ± 0.4 cm (range: 2.0–3.0 cm). In 3 cases (13.6%), transient attenuation of the Doppler signal was observed during maximal compression, which resolved immediately after slight (0.5 cm) back‐distraction. No case required abandonment of the S‐L technique due to irreversible signal loss or clinical signs of ischemia. This confirms the feasibility and safety of the 3‐cm Doppler‐guided threshold in this cohort. In terms of perioperative indicators, the WHT in the S‐L group (68.5 ± 25.2 days) was significantly shorter than that in the BT group (85.2 ± 21.5 days), and the difference was statistically significant (*p* < 0.05). Similarly, the BHI (37.36 ± 10.73 days/cm) and EFI (45.36 ± 11.23 days/cm) in the S‐L group were significantly lower than those in the BT group (44.14 ± 11.12 and 53.52 ± 12.55 days/cm), with statistically significant differences (all *p* < 0.05) (Table 2).

**TABLE 2:** Clinical results in the S‐L group and BT group.

Variable	S‐L group (*n* = 22)	BT group (*n* = 21)	*p*
WHT (days)	68.5 ± 25.2	85.2 ± 21.5	0.031
BHI (days/cm)	37.36 ± 10.73	44.14 ± 11.12	0.047
EFI (days/cm)	45.36 ± 11.23	53.52 ± 12.55	0.030
Complication‐related hospital stay (days)	8.2 ± 4.1	10.5 ± 5.3	0.125

Abbreviations: S‐L, shortening‐lengthening; BT, bone transport.

### 3.3 Complications

No deep infection, neurovascular injury, compartment syndrome, or amputation occurred during the surgery. Postoperatively, pin tract infection occurred in six patients in the S‐L group and five cases in the BT group, all of which were cured after dressing changes and oral antibiotic treatment. Nonunion at the docking site occurred in three cases in the S‐L group and four cases in the BT group, all requiring repeated bone grafting surgery. Two patients in each group developed ankle joint stiffness, and functional recovery was achieved after surgical release combined with rehabilitation exercises. In addition, one case of LLD occurred in the S‐L group, while none occurred in the BT group. According to the Paley classification criteria, there were two cases of axial deviation in the S‐L group postoperatively; whereas the BT group had three cases of axial deviation, mainly presenting as tibial varus deformity. There was no statistically significant difference in the overall complication rate between the S‐L group (45.5%, 14/22 cases) and the BT group (42.9%, 14/21 cases) (*p* > 0.05). Further analysis of severity according to the Paley complication classification criteria showed that the distribution of complications in the S‐L group was: Problems in six cases (27.3%), Obstacles in seven cases (31.8%), and Sequelae in one case (4.5%); while the BT group had five cases (23.8%) of Problems, nine cases (42.9%) of Obstacles, and zero cases of Sequelae (Table 3).

**TABLE 3:** Complications of the S‐L and BT groups.

Variable	S‐L group	BT group	*p*
Deep infection	0 (0%)	0 (0%)	
Axial deviation	2 (9.1%)	3 (14.3%)	
Docking site nonunion	3 (13.6%)	4 (19.0%)	
Pin tract infection	6 (27.3%)	5 (23.8%)	
LLD	1 (4.5%)	0 (0%)	
Joint stiffness	2 (9.1%)	2 (9.5%)	
Regenerate fracture	0 (0%)	0 (0%)	
Neurovascular injury	0 (0%)	0 (0%)	
Category			0.704
Problems	6 (27.3%)	5 (23.8%)	
Obstacles	7 (31.8%)	9 (42.9%)	
Sequelae	1 (4.5%)	0 (0%)	
Total patients affected	10	9	
Complications	10 (45.5%)	9 (42.9%)	0.864

Abbreviations: BT, bone transport; LLD, limb length discrepancy; S‐L, shortening‐lengthening.

Among complications requiring reoperation (Obstacles: 7 in S‐L, 9 in BT), docking site nonunion accounted for the majority, with a mean of 1.3 revision procedures per affected patient in the S‐L group and 1.5 in the BT group. The mean total hospital stay due to complication management was 8.2 ± 4.1 days in the S‐L group and 10.5 ± 5.3 days in the BT group (*p* = 0.125) (Table 2). No major complications (e.g., amputation, permanent nerve injury) occurred in either group. All pin tract infections were mild and managed without hospitalization.

### 3.4 ASAMI Bone Healing and Lower Limb Function Evaluation Grading

At the final follow‐up, evaluation was performed using the ASAMI criteria. In terms of bone healing evaluation, the S‐L group had excellent in 14 cases, good in 6 cases, and fair in 2 cases; the BT group had excellent in 14 cases, good in 5 cases, and fair in 2 cases, with no statistically significant difference (*p* > 0.05). In terms of functional evaluation, the S‐L group had excellent in 13 cases, good in 6 cases, and fair in 3 cases; the BT group had excellent in 12 cases, good in 7 cases, and fair in 2 cases, and there was no statistically significant difference between the two groups (*p* > 0.05) (Table 4).

**TABLE 4:** Results of ASAMI scores in the S‐L group and BT group.

Variable	Excellent	Good	Fair	Poor	*p*
Bone scores					0.993
S‐L group	14	6	2	0	
BT group	14	5	2	0	
Functional scores					0.872
S‐L group	13	6	3	0	
BT group	12	7	2	0	

Abbreviations: ASAMI, Association for the Study and Application of the Method of Ilizarov; BT, bone transport; S‐L, shortening‐lengthening.

Patients with ITBD‐STD were managed using either the S‐L or BT technique, both of which resulted in successful soft tissue reconstruction and bone union (A representative case is illustrated in Figures 2 and 3).

**FIGURE 2:**
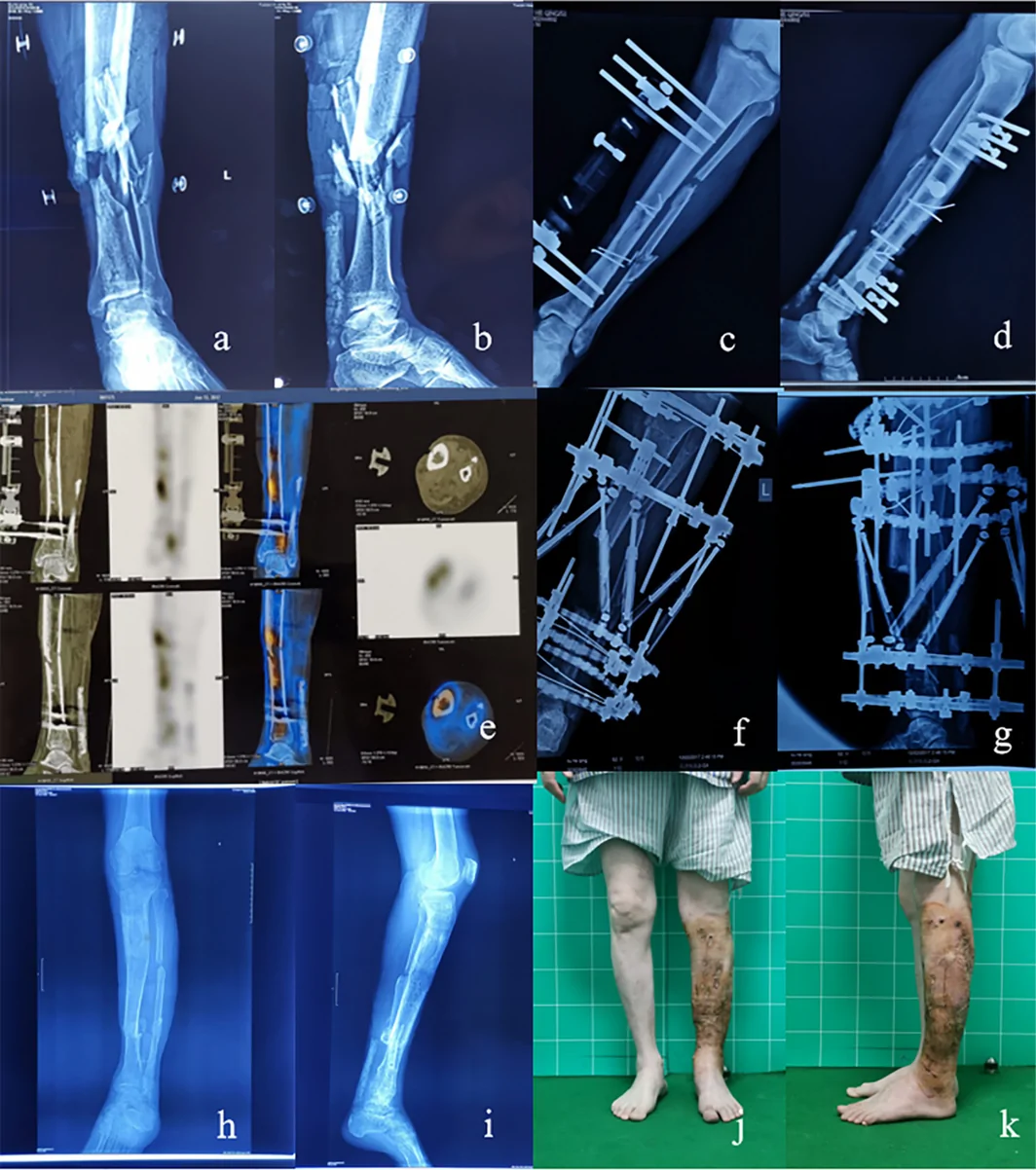
A 39‐year‐old male with ITBD‐STD was managed via the BT technique. (a, b) X‐ray images of the injured tibia at the time of injury. (c, d) Postoperative x‐ray showing unilateral external fixator fixation of the left tibia performed at an outside hospital emergency department. (e) SPECT/CT of the left lower leg showing increased bone metabolism in the middle and lower segments of the tibia, suggestive of an infectious lesion. (f, g) Postoperative x‐ray showing resection of the infected middle and lower tibial segment, fixation with a circular external fixator, and proximal tibial osteotomy. (h, i) Postoperative x‐ray showing external fixator removal, adequate mineralization at the bone lengthening site, and solid union at the docking site. (j, k) Clinical follow‐up photographs taken 1 month after removal of the circular external fixator.

**FIGURE 3:**
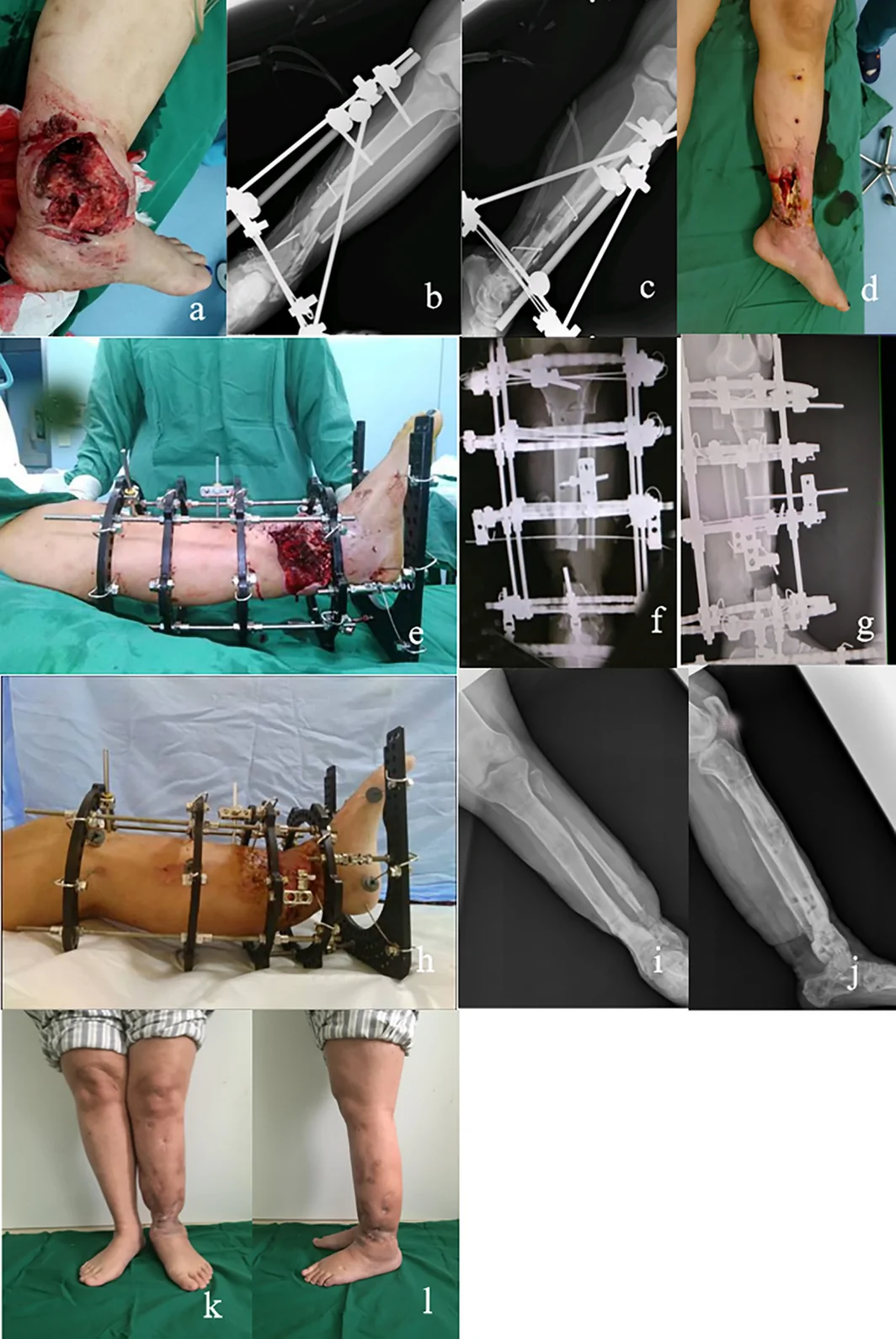
A 45‐year‐old female with ITBD‐STD treated with the S‐L technique. (a) Clinical photograph of the left lower leg at the time of injury. (b, c) Radiographs showing a comminuted tibial fracture fixed with a hybrid external fixator. (d) Clinical photograph demonstrating tibial exposure with an associated soft tissue defect. (e) Intraoperative photograph following debridement of the distal left lower leg, showing bone and soft tissue defects stabilized with a circular external fixator. (f, g) Postoperative radiographs showing resection of the infected middle and lower tibial segment with partial shortening, stabilized with a circular external fixator, and proximal tibial osteotomy. (h) Completion of left lower leg shortening‐lengthening with circular external fixation. (i, j) Postoperative radiographs demonstrating external fixator removal, adequate mineralization at the lengthening site, and solid union at the docking site. (k, l) Clinical follow‐up photographs taken 1 month after removal of the circular external fixator.

## 4 Discussion

This comparative study shows that for ITBD‐STD, the S‐L technique offers significant time‐related benefits over BT in terms of WHT, BHI, and EFI, yet these advantages do not translate into superior ASAMI functional scores or a lower overall complication rate. Given that final functional outcomes are comparable, the clinical value of S‐L hinges essentially on an objective safety framework for acute shortening. This study fills this gap by establishing a reproducible, Doppler‐guided protocol with a predefined 3‐cm safety limit and a transparent decision strategy. Unlike previous reports relying on subjective assessments without standardized endpoints [7, 13], our protocol provided a real‐time “signal‐present” safety lock. In our cohort, all 22 S‐L patients underwent shortening under continuous Doppler monitoring, averaging 2.8 ± 0.4 cm, confirming the clinical feasibility of this threshold.

### 4.1 Core Innovation: Validation of the Doppler‐Guided Safety Threshold and Treatment Selection Strategy

Building on this framework, validation of the 3‐cm threshold is key. In our series, shortening reached a mean of 2.8 ± 0.4 cm (range: 2.0–3.0 cm). Transient Doppler attenuation occurred in three cases (13.6%) at maximal compression but recovered instantly upon 0.5‐cm back‐distraction, and no case required S‐L abandonment. This confirms 3 cm as a safe, reproducible intraoperative endpoint.

This limit is anatomically grounded, preventing excessive vessel buckling and nerve traction in infected legs where soft tissue fibrosis often renders pedal pulses nonpalpable. Notably, in several patients, Doppler signals remained audible despite absent palpable pulses, underscoring the critical advantage of objective over subjective monitoring.

This validated safety framework removes vascular compromise as a confounding variable. Therefore, the observed advantages of S‐L (shorter WHT and EFI) can be more confidently attributed to the inherent design of acute shortening, rather than differences in intraoperative vascular management, setting the stage for the detailed comparisons in the following subsections.

### 4.2 Comparison of Efficacy Indicators and Prognosis Between the S‐L Group and the BT Group

The core finding of this study is that the WHT in the S‐L group was significantly shorter compared with the BT group (68.5 ± 25.2 vs. 85.2 ± 21.5 days, *p* < 0.05). Although the BHI (37.36 ± 10.73 vs. 44.14 ± 11.12 days/cm) and EFI (45.36 ± 11.23 vs. 53.52 ± 12.55 days/cm) were also significantly lower in the S‐L group (all *p* < 0.05), these ratio indices must be interpreted with extreme caution. Contrary to a possible concern, the denominator for BHI and EFI is identical in both groups (the original defect length), because the S‐L group's postoperative lengthening compensates for the acute shortening without adding extra distance beyond the original defect length. Thus, no mathematical bias exists. Therefore, the absolute reduction in WHT and overall treatment duration—rather than these ratio indices—more accurately reflects the primary advantage of the S‐L technique in reducing patient burden. Furthermore, there were no statistically significant differences between the two groups regarding the overall complication rate (45.5% vs. 42.9%, *p* > 0.05) or ASAMI functional scores (*p* > 0.05).

We acknowledge that the shorter treatment times in the S‐L group are largely inherent to its design—acute shortening directly facilitates early wound closure. This does not reflect an unanticipated biological advantage. Hence, we avoid claims of “superiority” and instead describe the S‐L technique as a clinically feasible option that reduces treatment duration when indicated, without compromising functional outcomes. Furthermore, the comparable ASAMI functional outcomes between groups temper any claim of overall superiority of the S‐L technique. Thus, our conclusion emphasizes time‐related benefits only, not functional advantage.

### 4.3 Comparison of Technical Characteristics and Clinical Applicability Between S‐L and BT

The bone S‐L technique creates favorable conditions for early closure or simplified wound coverage surgery by acutely shortening the bone defect site and directly bringing the soft tissue edges closer together [6, 15]. Its possible mechanisms include: (1) Reducing soft tissue tension: Acute shortening eliminates the continuous traction on the skin and subcutaneous tissues caused by the defect gap, reducing the difficulty of direct closure and decreasing reliance on complex flap surgeries. (2) Promoting early bone end contact: Although gradual lengthening is required subsequently, the early direct contact of bone ends may initiate the biological process of bone healing and create a better initial environment for final firm healing. However, we acknowledge that these theoretical mechanisms were not directly verified by histological or biomechanical data in our clinical cohort and should be interpreted as speculative. In contrast, the traditional BT technique prioritizes limb length preservation and repairs the defect by slowly pulling and transporting the bone segment. Its treatment course is more prolonged, and the external fixator wearing time is usually longer, imposing significant psychological and life burdens on patients [16]. The lengthy transport period also implies a higher risk of pin tract infection and a higher incidence of nonunion at the docking site [17]. In terms of patient tolerance, the S‐L technique may be associated with better patient compliance, but this remains to be confirmed with dedicated compliance or PROM assessments; whereas the BT technique imposes higher demands on patient endurance due to the longer external fixation time and complex adjustments.

### 4.4 The Critical Role of Vascular and Nerve Protection in the S‐L Technique

The safety of blood vessels and nerves is the absolute prerequisite for the S‐L technique. In this study, the establishment of a strict, real‐time Doppler‐guided protocol (≤ 3 cm) represents the core methodological advancement. Unlike previous studies that vaguely stated “without neurovascular compromise” [7, 12, 13], our protocol provided an objective “signal‐present” endpoint, mitigating both the risk of overly conservative shortening and the danger of catastrophic ischemia. This is particularly critical in infected tibial defects, where soft tissue fibrosis and edema often render traditional pedal pulse palpation unreliable—a phenomenon we observed intraoperatively in several patients, where Doppler signals remained audible despite non‐palpable pedal pulses. In our cohort, all 22 patients were safely shortened within the predefined 3‐cm protocol limit (mean 2.8 ± 0.4 cm) without irreversible Doppler signal loss, validating this objective threshold as a reproducible safety lock.

Beyond vascular monitoring, we implemented a systematic strategy for neural protection: (i) intraoperative acute shortening was generally capped at 3 cm; for larger defects, progressive shortening (1 mm per session, three times daily) was adopted postoperatively to provide a buffer for neurovascular adaptation [18]; and (ii) foot sensation and motor function were closely monitored, with immediate cessation of shortening upon any abnormality [19]. The combined application of these objective (Doppler) and clinical (neurological) monitoring measures demonstrates that acute shortening in the infected lower leg environment is both safe and feasible, offering surgeons a standardized framework that subjective examination alone cannot provide.

### 4.5 Management of the Docking Site and Promotion of Bone Healing

The bone S‐L technique may confer potential benefits for the healing of the docking site. The final bone end docking is completed during the lengthening phase, but the early acute shortening brings the bone stumps closer together, and there may already be some contact or apposition before lengthening begins. This early bone end contact may promote the formation of a local hematoma and the generation of initial callus tissue, initiating positive signals for bone healing during the subsequent lengthening process and after docking [20]. In contrast, the docking site in the BT technique is completely separated before transport and requires contact only after a long period and long‐distance migration; the blood supply at the docking site may be poorer, and the risk of soft tissue interposition is higher, which may be one of the reasons for its slightly higher rate of docking nonunion [21]. In this study, the docking site nonunion rate was 13.64% (3/22) in the S‐L group and 19.05% (4/21) in the BT group. Although the difference did not reach a statistically significant level, the trend is consistent with the above inference, but this finding must be confirmed with larger samples. All cases of docking site nonunion were successfully cured through debridement or bone grafting surgery. We note that these proposed mechanisms are speculative, as no histological or basic science validation was performed in this clinical study.

### 4.6 Analysis of Complications and Prevention Strategies

Pin tract infection is the most common complication of external fixator treatment. In this study, the incidence rates in the S‐L group and BT group were 27.3% and 23.8%, respectively, which is consistent with previous literature reports [10, 17]. All cases were controlled through intensified dressing changes, local care, and oral antibiotics, suggesting that strict pin tract care is a fundamental preventive measure throughout the entire treatment course.

In terms of axial deviation, although there was no difference in the overall complication rate, the BT group had three cases of tibial varus deformity, while the S‐L group had only two cases. This may be because during the BT process, the sliding bone segment is prone to alignment deviation due to factors such as uneven traction force and uneven soft tissue tension when moving over a long distance within the track [22, 23]; whereas the S‐L technique mainly adjusts at the defect plane, and axial control at the osteotomy and lengthening end is relatively easier. For the axial deviation that occurred, the deformities were effectively corrected after adjustment surgery with the external fixator. Importantly, the final removal of the external fixator strictly adhered to our radiological criteria (bridging callus in at least three cortices), ensuring that no residual mechanical axis malalignment compromised the patients' ability to achieve full, painless weight‐bearing. Regarding docking site nonunion, there were three cases (13.6%) in the S‐L group and four cases (19.0%) in the BT group, all requiring repeated bone grafting surgery. Although there was no statistically significant difference in incidence between the two groups, this remains an important factor affecting the treatment process, suggesting that sclerotic bone at the docking end needs to be thoroughly debrided intraoperatively, and close attention should be paid to observing callus growth postoperatively [20].

However, it is worth noting that although the S‐L technique is more conducive to wound closure and thus avoids flap repair, the tendons become slack after shortening, making foot drop prone to occur [14]. In addition, with the increase in the magnitude of bone lengthening by the external fixator, the tension on the external fixator pins and skin tendons increases, and the range of motion of adjacent joints gradually decreases [10]. Based on the experience of this study, for patients with pre‐existing foot drop deformity, combining pin fixation and correction of the foot and ankle while implementing the Ilizarov technique can effectively correct the deformity or prevent its further aggravation.

The incidence of ankle stiffness was similar between the S‐L group and the BT group, with two cases (9.09%) in the S‐L group and two cases (9.52%) in the BT group. This result may be related to the active functional exercises performed by patients in both groups postoperatively. Early initiation of functional exercises for the knee, ankle, and quadriceps muscles after surgery helps prevent the occurrence of joint stiffness. Complications such as LLD, neurovascular injury, and refracture were relatively rare in this study. There was no statistically significant difference in the overall complication rate between the two groups, which is consistent with the literature conclusion that there is no significant difference in complications between the two methods [17]. The one case of LLD in the S‐L group was managed by wearing shoe lifts.

We acknowledge that the current analysis primarily focuses on complication frequency rather than severity or clinical burden. However, the reoperation rate (Obstacles) and additional hospital days described above provide a preliminary assessment of the differential burden between the two techniques.

### 4.7 Comparison With Previous Studies and Novel Contributions of the Present Work

To highlight the unique contributions of the present study relative to previously published comparative series on S‐L versus BT for infected tibial defects, we summarized the key methodological and clinical features of the major studies in Table 5.

**TABLE 5:** Comparison of key study features between the present study and previously published comparative studies on S‐L versus BT for infected tibial defects.

Feature	Sigmund et al. [7]	Huang et al. [12]	Huang et al. [13]	Present study
Study design	Prospective	Retrospective	Retrospective	Retrospective
Sample size (S‐L/BT)	20/27	21/26	32/36	22/21
Specific techniques evaluated	ASR/BT	ASDL/BT	ASR/ACSBT	S‐L/BT
Mean bone defect (cm) (S‐L/BT)	4.0/5.9	8.9/10.3	6.5/7.1	6.2/5.4
Acute shortening limit/monitoring	Not specified (only “without neurovascular compromise”)	Not specified (only “circulation observed closely”)	Not specified (only “observe circulation”)	≤ 3 cm, real‐time Doppler‐guided
Clear technique selection strategy reported	Partial (defect ≤ 5 cm for S‐L)	No	No	Yes (explicit criteria, Section 2.1)
Denominator for BHI/EFI explicitly defined	No	No	No	Yes (original bone defect length, excluding acute shortening)
Complication burden quantified	Reoperation rate only	Obstacles rate only	Number of complications per patient	Reoperation rate + complication‐related hospital days
EFI (S‐L/BT)	2.0/1.8 months/cm (*p* = 0.223)	1.04/1.91 months/cm (*p* < 0.001)	1.5/1.4 months/cm (*p* > 0.05)	45.36/53.52 days/cm (*p* < 0.05)
Main conclusion on S‐L vs. BT	S‐L is superior in safety (fewer unplanned surgeries, especially docking site revisions), though it does not reduce EFI compared to BT.	S‐L is superior to BT, significantly reducing treatment duration (time in frame, EFI) and complication rates (obstacles).	Comparable in EFI and functional outcomes; Modified BT is superior to S‐L in controlling infection‐related complications, making it preferable for infected defects	S‐L reduces WHT and EFI, but offers no functional superiority; differences are design‐inherent.

Abbreviations: ACSBT, antibiotic calcium sulfate‐loaded bone transport; ASDL, acute shortening and double‐level lengthening; ASR, acute shortening and relengthening; BHI, bone healing index; BT, bone transport; EFI, external fixation index; S‐L, shortening‐lengthening; WHT, wound healing time.

## 5 Strengths and Limitations

This study has several notable strengths in its design and methodology. Technically, we established an explicit, Doppler‐guided safety threshold (≤ 3 cm) and a transparent decision strategy for choosing between S‐L and BT, which addresses the ambiguity in technique selection found in previous literature. Methodologically, we clarified the denominator definition for BHI and EFI (using the original bone defect length) to prevent mathematical bias, and we quantified the complication burden using reoperation rates and additional hospital days, moving beyond simple categorical reporting. Conceptually, by framing the S‐L technique's benefits strictly as time‐related and design‐inherent rather than evidence of biological superiority, we provide a more objective and clinically sound interpretation of the outcomes. However, this study also has several limitations. First, the retrospective study design may introduce selection bias; although there were no significant differences in baseline characteristics between the two groups, non‐randomized grouping may affect the reliability of the results. Moreover, no multivariable regression analysis was performed to adjust for potential confounders (e.g., defect size, soft tissue condition, vascular status), which further limits causal inference. Second, the relatively small sample size (43 cases in total) substantially limits statistical power. This is especially critical for complication‐related outcomes, where the wide confidence intervals and low post hoc power (e.g., < 0.20 for most complication comparisons) mean that the lack of statistically significant differences between groups cannot be taken as evidence of equivalence. Furthermore, the non‐significant *p*‐values for baseline characteristics (Table 1) do not guarantee that the two groups were truly comparable; unmeasured confounders, such as subtle differences in soft tissue viability, microvascular status, or inherent surgeon preference, may still exist. Third, although the Paley classification was used to categorize complications, the analysis remained descriptive. Due to the small absolute number of events, we did not perform a severity‐weighted scoring system or time‐dependent modeling, which limits the quantitative interpretability of the complication burden. Fourth, the follow‐up duration was relatively short (average 19.9 months), which is insufficient to assess late complications such as refracture of the regenerate bone, recurrent infection, or long‐term functional deterioration. The authors are continuing to follow this patient cohort and will report long‐term (≥ 5 years) functional and radiographic outcomes in a separate study. Future studies with larger sample sizes, prospective, randomized controlled designs, and long‐term follow‐up are necessary to further verify the conclusions of this study and provide higher‐level evidence support for clinical practice. Fifth, this study did not include any patient‐reported outcome measures (PROMs), such as quality‐of‐life or treatment burden questionnaires. Although we discuss patient tolerance, psychological burden, and quality‐of‐life implications in the Discussion, these statements are not supported by direct PROM data and should be considered speculative. Sixth, while functional recovery and joint mobility were systematically evaluated using ASAMI criteria, this study lacked objective, instrument‐based quantification of specific muscle strengths (e.g., isokinetic testing), relying instead on the clinical surrogate of the patient's ability to achieve full weight‐bearing. Future studies should incorporate validated PROMs to better capture the patient's perspective.

## 6 Conclusion

Both the S‐L technique and BT technique are effective in treating ITBD‐STD, with comparable safety profiles despite differing indications. When performed under the strict Doppler‐guided protocol (≤ 3 cm), the S‐L technique offers significantly shorter WHT without increasing complication rates; however, this time‐related benefit is largely inherent to the acute shortening design rather than evidence of an unanticipated biological advantage. Given that functional outcomes (ASAMI scores) are comparable between the two groups, the S‐L technique provides no functional superiority over BT. Nevertheless, the explicit Doppler criteria and the selection strategy established in this study provide a safe, reproducible framework for technique selection, validating the clinical feasibility of acute shortening without compromising final function or safety.

## Author Contributions

**Zhiming Zhao:** writing – original draft, investigation. **Guoqi Ji:** data curation, formal analysis. **Chengkuo Cai:** data curation, formal analysis. **Weiguo Xu:** supervision, writing – review and editing.

## Funding

This study was supported by Tianjin Natural Science Foundation (24JCYBJC01440).

## Ethics Statement

This study protocol was reviewed and approved by the Ethics Committee of Tianjin Hospital, Tianjin University (approval no. 2026‐002). All participants provided written informed consent for the use of their clinical data.

## Conflicts of Interest

The authors declare no conflicts of interest.

## Data Availability

The data that support the findings of this study are available on request from the corresponding author. The data are not publicly available due to privacy or ethical restrictions.
